# A 12-week multicomponent exercise program enhances frailty by increasing robustness, improves physical performance, and preserves muscle mass in older adults with HIV: MOVIhNG study

**DOI:** 10.3389/fpubh.2024.1373910

**Published:** 2024-04-17

**Authors:** Fátima Brañas, Jorge Díaz-Álvarez, Jesús Fernández-Luna, Brian D. Vásquez-Brolen, Rafael García-Molina, Elena Moreno, Pablo Ryan, Javier Martínez-Sanz, Laura Luna, Marta Martínez, Fernando Dronda, Matilde Sánchez-Conde

**Affiliations:** ^1^Geriatrics Department, Hospital Universitario Infanta Leonor, Madrid, Spain; ^2^FIIB H.U Infanta Leonor y H.U. Sureste, Madrid, Spain; ^3^Medicine Department, Universidad Complutense, Madrid, Spain; ^4^Infectious Diseases Department, Hospital Universitario Ramón y Cajal. IRYCIS, Madrid, Spain; ^5^Primary Care Service, Centro de Salud Fuentelarreina, Madrid, Spain; ^6^Geriatrics Department, Hospital Nuestra Señora del Perpetuo Socorro, Albacete, Spain; ^7^CIBER de Envejecimiento y Fragilidad (CIBERFES), ISCIII, Madrid, Spain; ^8^CIBER de Enfermedades Infecciosas (CIBERINFEC). ISCIIII, Madrid, Spain; ^9^HIV Clinic, Hospital Universitario Infanta Leonor, Madrid, Spain

**Keywords:** HIV, older adults, exercise, frailty, physical performance, muscle mass

## Abstract

**Background:**

Our aim was to analyze the effects of a multicomponent exercise program (MEP) on frailty and physical performance in older adults with HIV (OAWH) since exercise can reverse frailty in the older population overall, but there is no data for OAWH.

**Methods:**

A prospective longitudinal study with intervention and control group was designed. Sedentary adults 50 or over with and without HIV were included. The intervention was a 12-week home-based MEP. Dependent variables were frailty (frailty phenotype), physical performance (Senior Fitness Test), muscle mass (ASMI) by bioimpedance. Pre- and postintervention measurements were analyzed using McNemar’s test for categorical variables and the Wilcoxon signed-rank test for quantitative variables.

**Results:**

40 OAWH and 20 OA without HIV. The median age was 56.5 years. 23.3% were women. The prevalence of frailty was 6.6% with no frail HIV-negative participants. Three of the four frail HIV-participants transitioned two (50%) from frail to prefrail and one (25%) to robust after the MEP. In participants with an adherence ≥50%, physical performance was significantly improved [basal vs. 12 week]: upper extremity strength [13 (13–15) vs. 16 (15–19), *p* = 0.0001], lower extremity strength [13 (11–16) vs. 15 (13–16), *p* = 0.004], aerobic endurance [62 (55–71) vs. 66 (58–80), *p* = 0.005]. Participants with low adherence experienced a significant worsening in ASMI [8.35 (7.44–9.26) vs. 7.09 (6.08–8.62), *p* = 0.03].

**Conclusion:**

A 12-week MEP enhances frailty by increasing robustness in OAWH, and improves physical performance, and preserves muscle mass in older adults with good adherence to the MEP independently of HIV status.

**Clinical trial number: NCT 05435521:**

## 1 Introduction

Aging is a success for people living in developed countries but, from the individual point of view, aging leads to a loss of function not only at a molecular, cellular or organ level but also of physical and cognitive function with a negative impact on quality of life and autonomy (1, 2). Considering health and social systems, the aging of the population is a costly issue in terms of the consumption of limited resources mostly due to the burden of disease and disability accompanying the aging process if appropriate preventive measures are not deployed. Moreover, aging with HIV has become achievable through the implementation of antiretroviral treatment, but it presents an even greater challenge in ensuring autonomy, maintaining quality of life, and preventing disability.

Although training programs for older adults cannot stop the biological aging process, they have the potential to mitigate the impact of aging on their physical performance (1). Several systematic reviews and meta-analyses have demonstrated that physical exercise is the gold-standard treatment for functional improvement in older adults (3, 4). In addition, multicomponent exercise programs (MEP) that include strength and power training have added benefits for cardiovascular and respiratory function, cognitive and affective status, reduced sarcopenia, falls and fear-of-falling syndrome and frailty in the overall population (5–7). Frailty is a common condition among older adults with HIV (OAWH) which is closely related to negative health outcomes, including mortality, but it is potentially reversible (8). Exercise, notably a MEP, is the only intervention that has been able to prove the reversion of frailty in the overall population (6), in a randomized controlled trial in one out of three frail patients (31.4%) (9). No data are available regarding exercise as treatment for frailty in OAWH. Data concerning exercise and HIV are scarce, with most studies primarily focusing on young individuals and exploring aspects such as alterations in body composition, cardiorespiratory function, and strength (10). Moreover, the investigations often focus on isolated components of the frailty phenotype rather than comprehensively studying the effect of exercise on the frailty phenotype itself. The World Health Organization (WHO) recommends at least 150–300 weekly minutes of moderate-intensity aerobic physical activity (PA) and strength training 2 or more days per week for people aged 65 years and older (11). Exercise should be prescribed as a treatment and/or preventive tool according to the physical condition of each individual considering type, time, and “dose” (frequency and intensity); however, in clinical practice, there is still a significant gap between these recommendations and actual implementation. Furthermore, exercise should be integrated in the daily routine of each older adult to generate a habit, because the maintenance of exercise is important for the persistence of its beneficial effects. Some complex and closely supervised exercise programs may have proven to be effective in the research setting, but their implementation in real life is very difficult. For all these reasons, the aim of our study is to know the effectiveness of a personalized MEP in real-life conditions in older adults with and without HIV on frailty and physical performance and, secondarily on quality of life, geriatric syndromes, and muscle mass.

## 2 Materials and methods

### 2.1 Design and participants

We performed a longitudinal, prospective, controlled, and multicenter study with intervention and follow-up at 12, 24, and 48 weeks. The current work shows the results of the study at 12 weeks. We enrolled sedentary adults with and without HIV, 50 years or older, according to the WHO’s definition of sedentarism (11). Participants with HIV were recruited through systematic sampling, offering participation in the study to all individuals who attended the HIV clinics of the Hospital Universitario Ramón y Cajal and the Hospital Universitario Infanta Leonor in Madrid (Spain) from November 4, 2021. Those who met all inclusion criteria, none of the exclusion criteria, and accepted to participate were included. Controls without HIV were selected from the HIV participants’ environment: partners, friends, or relatives to ensure similar sociocultural characteristics and were matched by age and sex. The recruitment concluded on February 15, 2022. The exclusion criteria included having any physical limitation that restricts walking and/or the development of an exercise session, undergoing steroid treatment, and/or suffering from some of the following diseases: acute myocardial infarction (recent 6 months) or unstable angina, uncontrolled atrial or ventricular arrhythmias, severe aortic stenosis, acute endocarditis or pericarditis, uncontrolled arterial hypertension (>180/100 mmHg), acute thromboembolic disease, or severe acute heart failure. The participation in the study was voluntary, and a written informed consent was obtained from all the participants. The ethics committees of the two hospitals participating in the study approved the study procedures.

The sample size was calculated considering the demonstrated efficacy of a MEP in reversing frailty by 30% (9) and the demonstrated ability of a MEP to reverse pre-frailty by 50% in the older overall population with a significance level of *α* = 0.05 and an effect size of 0.2. Considering a 50% loss rate since, in this study the results are shown at 12 weeks, but the study extends for 12 months, the estimated sample size was 40 participants, 20 in each group. Finally, we recruited 60 participants, 20 more participants with HIV than necessary to ensure reaching a proportion of frail participants representative of the frailty prevalence within the HIV population.

### 2.2 Data collection

At baseline, sociodemographic characteristics (sex assigned at birth), HIV-related data, and medications were recorded. Self-reported comorbidities were also registered from the following: hypertension, type 2 diabetes, dyslipidemia, COPD, chronic kidney disease, cancer (<5 years from diagnosis), history of cancer (≥5 years from diagnosis; not active disease), psychiatric disease, liver disease, and osteoarticular disease. In every visit, frailty, physical function, physical performance, geriatric syndromes, patient-reported outcome measures (PROMS), and dietary and physical habits were documented. Muscle mass was measured using the appendicular skeletal muscle mass index (ASMI) by electrical bioimpedance (Tanita BC-418^®^).

### 2.3 Outcomes measurements

Frailty was defined according to Fried’s criteria (12), namely shrinking (unintentional weight loss of ≥4.5 kg or ≥5% of body weight during the previous year), weakness (grip strength adjusted for sex and BMI), poor endurance and low energy (self-reported exhaustion identified by two questions from the Center for Epidemiologic Studies Depression scale), slowness (based on time to walk 4·6 m, adjusting for gender and standing height), and low PA level (<383 kcal/week in men and <270 kcal/week in women using the Minnesota Leisure Time Activity Questionnaire). Patients were considered frail if they met at least three of the five criteria, prefrail when they met one or two criteria, and robust when they met no criteria. Reversibility of frailty was defined as a change in frailty status from frailty to prefrailty or robustness and/or from prefrailty to robustness after the intervention.

Improvement of physical function was assessed by objective measures of strength, gait speed, and balance using the Short Physical Performance Battery (SPPB) (13). Improvements in 0.1 m/s in gait speed and/or 1 point in the SPPB score after the intervention were considered significant. Improvement of physical performance, endurance and resistance fitness levels were assessed using the Senior Fitness Test (SFT) (14). SFT is composed by six different functional tests of strength, endurance, balance, agility, and flexibility which are scored separately on different scales. The six tests are the Chair Stand Test that reflects lower body strength, the Biceps Curl Test that reflects upper body strength, the 2-Minute Step Test to assess the aerobic endurance, the Chair Sit and Reach Test that reflects lower body flexibility, the Back Scratch Test to measure the upper body flexibility and the Up-and-Go test to evaluate agility and dynamic balance. Improvements in the SFT after the intervention were considered separately in the various functional tests of lower body strength, upper body strength, aerobic endurance, flexibility, agility, and dynamic balance. Improvement in PROMS was evaluated by comparing scores before and after the intervention. The patient’s health-related quality of life was measured by self-administered validated questionnaires such as the WHOQOL-HIV-BREF and the EQ-5D visual analog quality of life scale (15). To evaluate each patient’s mood, depression and/or anxiety status we used the self-administered validated questionnaire as the HADS Scale for depression and anxiety. Improvement in geriatric syndromes: falls, depression, and malnutrition. Falls were defined as any self-reported falls in the past year. Depression was tested using the Geriatric Depression Scale Short Form (GDS-SF) (16), and the risk of malnutrition was assessed using the Mini Nutritional Assessment Short Form (MNA-SF) (17), and by using objective measurements (weight, height, and body mass index). Changes in the prevalence of the geriatric syndromes after the intervention were registered.

Dietary habits were registered before and after the intervention through a dietary questionnaire and physical habits were documented in METS (metabolic equivalent of task) using the International Physical Activity Questionnaire (IPAQ) (18). Changes in muscle mass were evaluated by comparing the parameters obtained from the bioimpedance measurements at all visits.

### 2.4 Intervention: personalized multicomponent exercise program

The intervention was a personalized MEP including strength, endurance, balance, and flexibility training performed in real-life conditions. The MEP features are described below following the Consensus Exercise Reporting Template (CERT) listed under 7 domains: (19).

1. What (material required). Basic material (mat, elastic band, and activity bracelet) was needed, which was provided at the baseline visit. Elastic bands were 5 kg-, 15 kg-, and 25 kg-resistance depending on the intensity of the program.
2. Who (provider). The MEP was designed by a Ph.D. in physical activity and sports, and it was explained and shown to the participants in two live sessions by a qualified personal trainer who was a registered nurse too. He provided the coaching during the follow-up.
3. How (delivery). Exercise was performed individually. The exercise program was preceded by instruction by the personal trainer in two face-to-face sessions. Before the study commenced, a study website was created where each participant had a private profile and could access to his/her exercise program, warm-up and stretching exercises, the schedule of visits and a contact area with the trainer and the research team. Each exercise in the web was accompanied by a video in which the trainer performs and explains it. We also provided QR cards to facilitate access to the program at any place and at any time. The trainer, by means of remote supervision, proactively contacted each participant on a weekly basis to supervise the proper compliance with the exercise program. The participants also had the option to contact the trainer via WhatsApp and/or email at any time to raise doubts about the program’s performance and/or report incidents such as the onset of pain or any other issue affecting the program’s development, with the commitment of a response within 24 h from the trainer (via WhatsApp, email, or a call if necessary), who explained and/or adapted exercise as needed. WhatsApp or mail (patient’s choice) were used as motivation strategies too. An important part of the intervention was that the qualified trainer also sent weekly personalized messages of encouragement, and stimulation to each participant to continue adhering to the plan. Other motivation strategies were developed. Before starting the program, during the recruitment visit, following the comprehensive assessment, the doctor dedicated time to motivate the participant by explaining the importance of adhering to the program, addressing logistical concerns, and explaining the open communication channels between the participant and the research team throughout the program’s development. During that visit, participants were also instructed on how the activity bracelet works and assisted in synchronizing it with their phone. The two sessions prior to the exercise program were used not only to teach participants technically how to do the exercises but also to motivate them to comply with the program.
4. Where (location). Strength, balance, and flexibility exercises were home-based. The aerobic exercise focused on outdoor walking. This is a MEP performed in real-life conditions with no non-exercise components.
5. When, how much (dosage). Four MEP programs were designed. The programs ranged from 1 to 4, progressively increasing the intensity, and the difficulty of the exercises. Each program consisted of three different circuits composed of seven exercises involving major muscle groups each: squats, shoulders – chest, lower body, gluteus, abdomen and back, and balance and flexibility. Each circuit was 45 min duration, 2–3 days per week for 12 weeks, 2 days were mandatory, and the third one was optional. Each exercise consisted of 2–3 series of 9–20 repetitions, depending on the program, with 45–60 s of inter-set rest. Before starting the strength, flexibility, and balance exercise circuits, the participant must perform the prescribed warm-up exercises, and at the end, the stretching exercises indicated. The endurance goal was defined considering the number of daily steps. For example, the aerobic goal in the first week of Program 1 was to walk 7,500 steps for 5 days a week and 10,000 steps for the other 2 days. As the difficulty of the program increases, changes in pace are prescribed in addition to increasing the step target. The complete MEP and the detailed description of each exercise to enable replication is freely available on the MOVIhNG App, both on Apple and Android.
6. Tailoring (what, how). As mentioned, four MEP programs were designed. The decision for determining the starting level of exercise was taken by the multidisciplinary team (doctor, nurse, Ph. D expert on exercise, and trainer) according to the results of the individual comprehensive assessment made and considering the physical habits (IPAQ), physical function (SPPB), physical performance (Senior Fitness Test) but also the mood status, preferences, self-confidence, and exercise predisposition of each participant. Exercises were tailored to the individual from the beginning if needed. Some examples: (1) participant with left shoulder tendinitis. The left shoulder was worked on but with less intensity. The exercise was adapted by instructing the patient to position their feet closer to the wall to perform a specific exercise, lift the elastic band upward only with the right shoulder and with the left, lift the sore shoulder without the elastic band until the point of no pain. (2) Participant with good physical function that would have allowed starting with program 2 but felt insecure because he has never exercised before. He was advised to start with program 1 and the coach increased monitoring. Once the participant adhered to the program and increased his confidence, he was transitioned to program 2. Moreover, we developed three different circuits for each program to avoid monotony and to provide the participants with multiple options to work the same muscle group, allowing them to choose the one that best suits their preferences and/or needs. The exercise program was designed to be carried out over 12 weeks during which progressive changes were established in the volume and intensity of exercise. Volume was defined as the total number of repetitions of each exercise and the number of times the circuit of the 7 exercises was completed. Intensity was defined as the load used (the resistance in kg of the elastic band), the reduction in rest time between exercises, and the maximum effort exerted through the Rate of Perceived Exertion (RPE), a scale that each participant recorded in the weekly activity diary on the website. By way of example, the progression established in Program 1 was as follows: weeks 1–2 (9–11 repetitions of each exercise/60 s rest between exercises/2 repetitions of the complete circuit of exercises); weeks 3–4 (11–12 rep/45 s/x 2); weeks 5–6 (13–15 rep/60 s/x 2); weeks 7–8 (13–15 rep/45 s/x 2); weeks 9–10 (11–12 rep/45 s/x 3), and weeks 11–12 (13–15 rep/45 s/x 3). It’s important to note that each participant was committed to filling out the weekly activity log on the website. With the recorded information including the RPE scale, data obtained from the activity bracelet regarding aerobic activity and transferred to the activity log, participant-reported compliance, and any potential issues communicated through established channels, the coach, with access to all this information, assessed weekly whether each participant was ready to continue with the established progression in each program or needed custom adjustments upward or downward in volume and/or intensity.
7. How well (adherence). To calculate adherence to the MEP, the following factors were considered: the number of sessions completed for strength, flexibility, and balance exercises, divided by the total number of scheduled sessions, which amounted to 24 sessions over 12 weeks; the number of days in which the step count recorded by the activity bracelet met the set goal, divided by the total number of days in the program, and the program adherence self-reported by the participant and recorded in the activity diary. The latter data allowed us to understand the degree of participant alignment with a mutually agreed-upon plan, as sometimes session compliance was correct in terms of quantity (volume), but the participant indicated that he could have exercised with greater intensity and/or diligence. For the total calculation of adherence to the MEP, adherence to strength, flexibility, and balance exercise sessions accounted for 60% of the total, adherence to aerobic exercise accounted for 30%, and self-reported adherence by the participant accounted for 10% of the total. Adherence was categorized as poor (<30%), fair (30–50%), moderate (50–80%), and good (>80%). However, we group it as poor if the compliance was less than 50% and good if the compliance was higher than 50% because the number of participants in each subgroup was small, and more importantly, based on the scientific evidence available within the overall population that has demonstrated the association between attending 50% of a MEP and the recovery of robustness among frail older adults (9).

### 2.5 Statistical analysis

The descriptive analysis of participants’ characteristics was performed using frequency distributions (median and 25th–75th percentiles). We used Fisher’s exact test for the comparison of categorical variables and the Wilcoxon rank-sum test for quantitative variables. We chose non-parametric tests due to small subgroup sizes and potential data distribution variability. Paired variables, specifically pre- and post-intervention measurements, were analyzed using McNemar’s test for categorical variables and Wilcoxon signed-rank test for quantitative variables. The analysis was per protocol. All statistical tests were two-tailed. All statistical analyses were performed using Stata v. 16.1 (StataCorp LP, College Station, TX, United States). Figures were created using GraphPad Prism v. 9.2 (GraphPad, La Jolla, CA, United States).

## 3 Results

**Figure 1:**
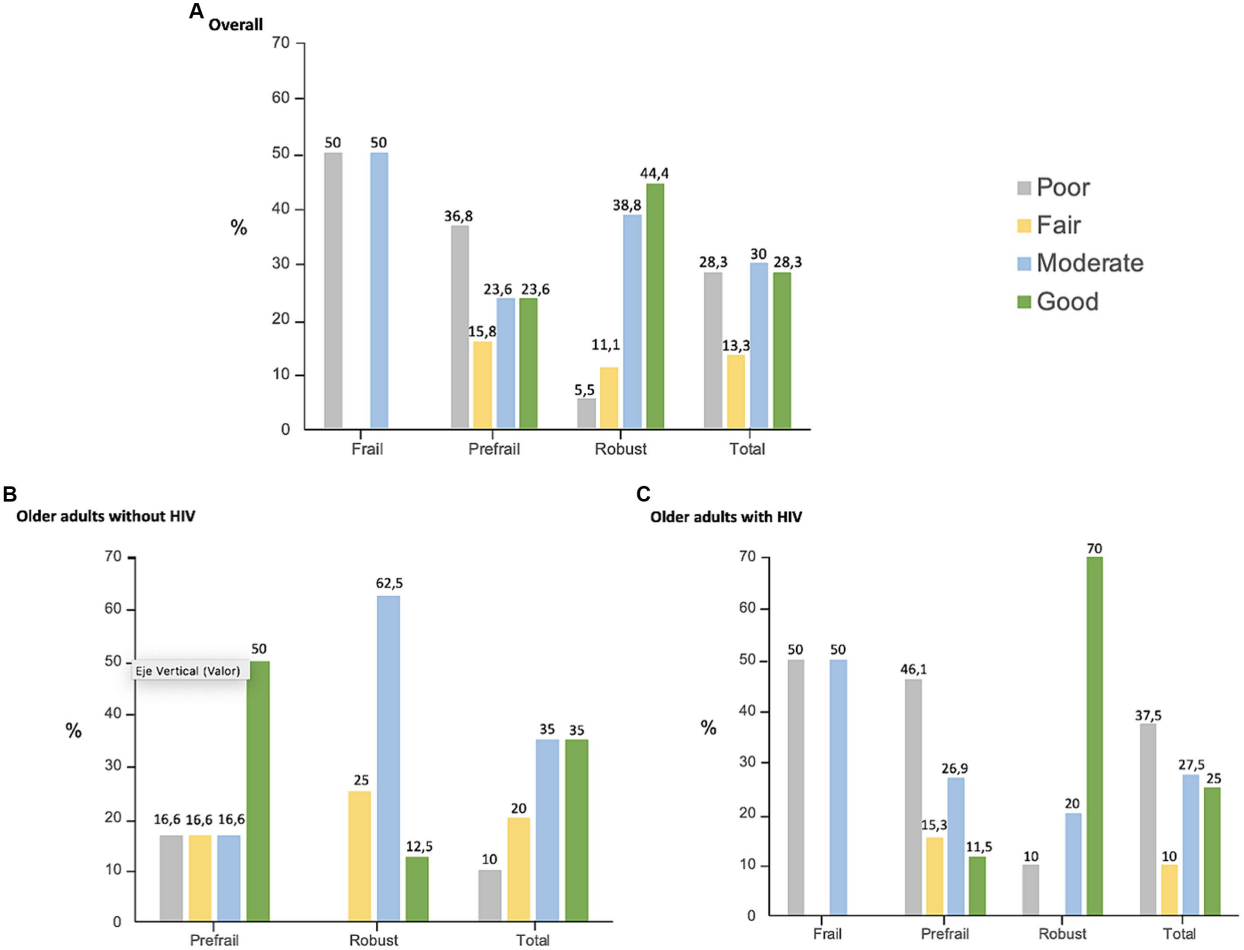
Adherence to the MEP according to the frailty status in the overall population and by HIV status. The *x*-axis represents the adherence to the 12-week MEP by frailty status in the overall population **(A)**, In older adults without HIV **(B)**, In OAWH **(C)**. The *y*-axis represents the percentage of participants with poor (<30%), fair (30–50%), moderate (50–80%), and good (>80%) adherence.

Sixty participants were included, 40 were OAWH and the controls were 20 older adults without HIV. The median age was 56.5 years, 23.3% were women, and half of them lived alone. Table 1 shows the baseline characteristics of all participants by HIV status and by the adherence to the MEP. Participants had a median of 3 (1–4) comorbidities, and good physical function. The prevalence of frailty was 6.6% with no frail participant in the HIV-negative group. The prevalence of falls was 21.6% with no differences according to the HIV status. All participants with HIV were virologically controlled, had a median of 19.7 (12–29.9) years with HIV, and injection drugs use was the route of HIV acquisition in 12.5%. Half of them were on CDC stage A and 22% on CDC stage C. Adherence to the MEP was described as poor (<50%) by 16 participants (26.6%), and good (>50%) by 35 participants (58%). We considered that the missing participants, nine (15%), had bad adherence <50%; therefore, 58.3% of the overall participants, 52.5% of the OAWH, and 70% of the OA without HIV had good adherence to the MEP. The adherence to the MEP according to the frailty status in the overall population and by HIV status is described in Figures 1A–C.

**Table 1:** Baseline characteristics by HIV status and by the adherence to the multicomponent exercise program.

	Total	HIV–	HIV+	Poor adherence to the MEP	Good adherence to the MEP
*N*	60	20	40	25 (41.67)	35 (58.33)
Sociodemographic characteristics, comorbidity, and medications					
HIV infection. *N* (%)	40 (66.7)			19 (76)	21 (60)
Sex assigned at birth. Female. *N* (%)	14 (23.3)	5 (25)	9 (22.5)	6 (24)	8 (22.9)
MSM. *N* (%)	25 (41.6)	4 (20)	21 (52.5)	13 (52)	12 (34.3)
Age. Years. Median (p25–p75)	56.5 (53–61.5)	58.5 (53–62.5)	56 (53–61)	55 (53–61)	57(53–62)
Living. *N* (%)					
Alone With partner Others	30 (50) 21 (35) 9 (15)	10 (50) 10 (50) 0	20 (50) 11 (27.5) 9 (22.5)	9 (36) 10 (40) 6 (24)	21 (60) 11 (31.4) 3 (8.6)
Comorbidity. Median (p25–p75) Specific comorbidities. *N* (%) Hypertension Diabetes Dyslipidemia COPD Chronic kidney disease Current cancer History of cancer Depression (on antidepressants) Liver disease Osteoarthritis	3 (1–4) 16 (26.6) 4 (6.6) 23 (38.3) 2 (3.3) 4 (6.6) 1 (1.6) 3 (5) 15 (25) 19 (31.6) 19 (31.6)	2 (1–4) 5 (25) 3 (15) 5 (25) 1 (5) 0 1 (5) 0 2(10) 3 (15) 5 (25)	3 (2–4) 11 (27.5) 1 (2.5) 18 (45) 1 (2.5) 4 (10) 0 3 (7.5) 13 (32.5) 16 (40) 14 (35)	3 (3–4) 9 (36) 3 (12) 13 (52) 1 (4) 4 (16) 0 1 (4) 6 (24) 10 (40) 7 (28)	2 (1–4) 7 (20) 1 (2.9) 10 (28.6) 1 (2.9) 0 1 (2.9) 2 (5.7) 9 (25.7) 9 (25.7) 12 (34.3)
Medications. Median (p25–p75) Polypharmacy*. *N* (%) Specific medications. *N* (%) Benzodiazepines Neuroleptics Hypnotics First line pain killers Opioids	3 (1–4) 10 (16.6) 13 (21.6) 1 (1.6) 4 (6.6) 11 (18.3) 6 (10)	2 (0–4) 1 (5) 0 0 0 0 1 (5)	3·5 (2–4) 9 (22.5) 13 (32.5) 1 (2.5) 4 (10) 11 (27.5) 5 (12.5)	4 (2–4) 5 (20) 8 (32) 1 (4) 2 (8) 6 (24) 4 (16)	2 (1–4) 5 (14.3) 5 (14.3) 0 2 (5.7) 5 (14.3) 2 (5.7)
Lifestyle					
Alcohol intake. *N* (%)	8 (13.3)	4 (20)	4 (10)	3 (12)	5 (14.3)
Current smoker. *N* (%)	19 (31.6)	6 (30)	13 (32.5)	11 (44)	8 (22.9)
Dietary habits**					
Meals a day. Median (p25–p75) Dairy. Median (p25–p75)	3 (3–4) 7 (5.5–13)	3 (3–4) 10 (7–14)	3 (3–4) 7 (5.5–13)	3 (3–4) 7 (4–11)	3 (3–4) 8 (7–14)
Physical Activity. METS Median (p25–p75)	396 (239.5–990)	528 (330–1113.7)	330 (115.5–924)	264 (0–330)	742.5 (320–1,188)
Physical function					
Hand grip strength. Kg Median (p25–p75)	33.2 (26.9–39)	32.8 (27–38.3)	33.2 (26.9–39.4)	32.3 (26.6–36.7)	36 (27.9–40.2)
Gait speed. m/s. Median (p25–p75)	1.19 (1.34–1.07)	1.19 (1.41–1.08)	1.19 (1.33–1.07)	1.17 (1.37–1.05)	1.20 (1.37–1.08)
SPPB score. Median (p25–p75)	11 (10.5–12)	11 (11–12)	11 (10–12)	11 (10–12)	11 (11–12)
Physical performance. Senior Fitness Test					
Lower limb strength. Chair Stand Test.^ Median (p25–p75)	13 (11–14.5)	13.5 (12–16)	12 (10.5–14)	13 (12–14)	13 (11–16)
Upper limb strength. Biceps Curl Test.^ Median (p25–p75)	13 (12–15)	13.5 (12.5–15.5)	13 (11.5–14.5)	13 (12–14)	13 (13–15)
Aerobic endurance. 2 min Step Test. Median (p25–p75)	59 (50–68.5)	65.5 (59.5–76)	56 (42.5–63.5)	56 (42–63)	62 (55–71)
Agility.TUG. sec. Median (p25–p75)	7.09 (6.2–8.1)	7.31 (6.2–8.4)	6.93 (6.2–7.9)	6.93 (6.09–8.16)	7.19 (6.28–8.05)
Frailty and geriatric syndromes					
Frailty. *N* (%)					
Robust Prefrail Frail	18 (30) 38 (63.3) 4 (6.6)	8 (40) 12 (60) 0 (0)	10 (25) 26 (65) 4 (10)	3 (12) 20 (80) 2 (8)	15 (42.9) 18 (51.4) 2 (5.7)
Falls^^. *N* (%)	13 (21.6)	4 (20)	9 (22.5)	5 (20)	8 (22.9)
Geriatric Depression Scale Short Form. Score. Median (p25–p75)	3 (1–5)	2 (1–4)	3.5 (1–6.5)	4 (1–7)	2.5 (1–5)
Nutritional status					
BMI. Median (p25–p75) MNA-SF. *N* (%) 8–11 12–14	26.5 (23–30.2) 11 (18.3) 49 (81.6)	26.7 (24.2–30.2) 2 (10) 18 (90)	26.3 (22.7–30.2) 9 (22.5) 31 (77.5)	27 (23–30.4) 5 (20) 20 (80)	25.4 (23.1–30.2) 6 (17.1) 29 (82.9)
Patient-reported outcome measures (PROMS)					
Not satisfied with his/her life. *N* (%)	17 (28.3)	2 (10)	15 (37.5)	6 (24)	11 (31.4)
HADS-anxiety score. Median (p25–p75)	6.5 (5–10.5)	6.5 (5.5–8.5)	6.5 (4.5–11.5)	6 (5–11)	7 (4–10)
HADS-depression score. Median (p25–p75)	3 (1.5–6)	2 (1.5–4)	4 (1.5–7)	4 (2–7)	3 (1–6)
WHO-QOL-HIV-BREF. Median (p25–p75)					
Physical Psychological	10.5 (9–12) 16.5 (14–18)	10 (8.5–11) 17 (15.5–18.5)	11 (9.5–12.5) 16 (13–18)	11 (9–12) 16 (15–18)	10 (9–11) 17 (13–18)
Muscle mass					
ASMI. g. Median (p25–p75)	7.92 (7.2–8.8)	8.05 (7.35–8.4)	7.8 (7.2–8.9)	8.35 (7.44–9.26)	7.88 (7.13–8.38)

Percentages are of the total for the column. MEP: multicomponent exercise program. Poor adherence: if the adherence to the 12-week MEP is <50%. Good adherence: if the adherence to the 12-week MEP is ≥50%. MSM: men who have sex with men. *Polypharmacy: defined as ≥5 concomitant medications excluding TAR. **Dietary habits: there were no vegans or vegetarians. There were no differences in the weekly consumption of legumes, potatoes, bread/cereals, fruit, vegetables, meat, fish, eggs, soft drinks, coffee or tea, beer, wine, or spirits. SPPB: short physical performance battery. ^Repetitions in 30 s. TUG: timed-up-and-go test. ^^Falls: defined as any self-reported falls in the past year. BMI: body mass index; MNA-SF: mini-nutritional assessment short-form; ASMI: appendicular skeletal muscle index.

**Figure 2:**
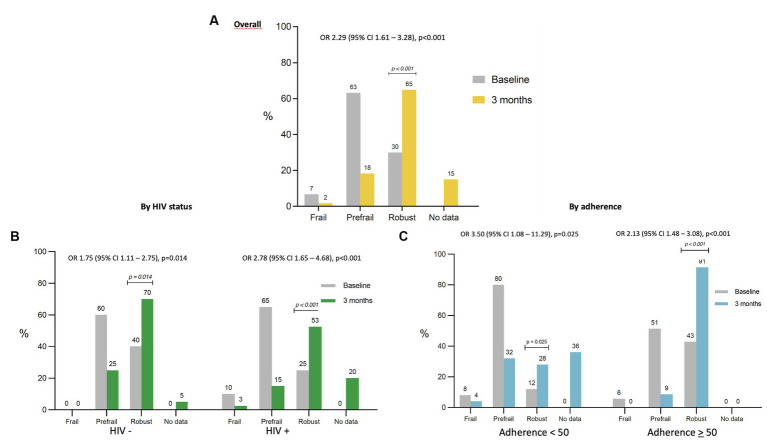
Risk of robustness after the 12-week MEP in the overall population, by HIV status, and by adherence to the MEP. The *x*-axis represents the frailty status at the baseline visit and after the 12-week MEP, in the overall population **(A)**, by HIV status **(B)**, and by adherence to the MEP **(C)**. The *y*-axis represents the percentage of participants in each status of frailty. The OR represents the odds of being robust after the 12-week MEP in the overall population **(A)**, by HIV status **(B)**, and by adherence to the MEP **(C)**.

The 12-week MEP significantly improved frailty. Out of the four frail participants at baseline, three improved their condition at 12 weeks, transitioning from frail to prefrail in two cases and to robust in one case. The other participant reported no adherence to the MEP and remained frail. Overall, the 12-week MEP significantly improved frailty interpreted as an increase in the percentage of robust participants. From 42 non-robust participants, 22 (52%) became robust after the 12 weeks MEP (Figure 2A). Therefore, the percentage of robustness increased from 30 to 65% with an odd ratio of 2.29 (95% CI 1.61–3.28). The improvement was independent of HIV status (Figure 2B). Participants adhering to the program achieved a very high percentage of robustness (91%) (Figure 2C), as is shown in Figure 2.

**Figure 3:**
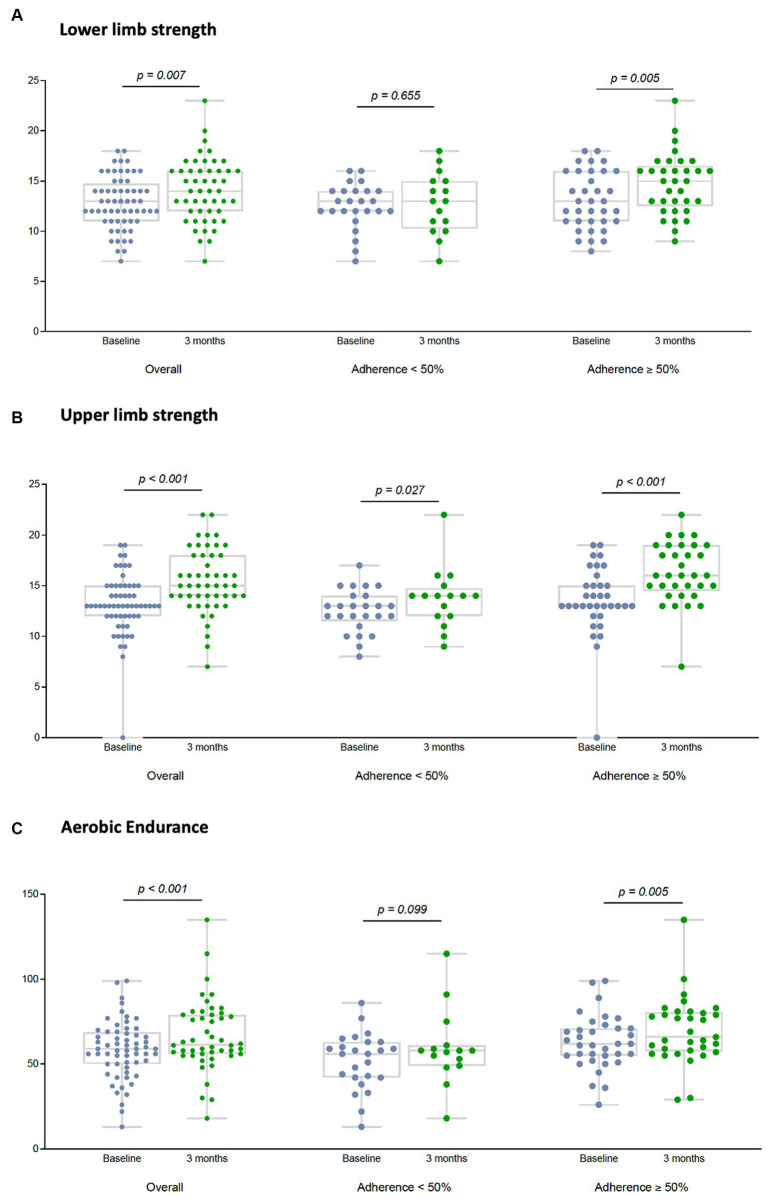
Effect of the 12-week MEP on physical performance by adherence to the MEP on older adults regardless of HIV status. Physical performance was defined considering lower limb strength and measured using the Chair Stand Test; upper limb strength was evaluated using the Biceps Curl Test; and aerobic endurance was registered through the 2 Minute Step Test. All three tests are part of the SFT. Lower limb strength (LLS) **(A)**, upper limb strength (ULS) **(B)**, and aerobic endurance (AE) **(C)** were represented by dot plots at the baseline visit and after the 12-week MEP, in the overall population, and by adherence to the MEP. Total number of participants were [basal 60 vs. 12 weeks 49]. Participants with adherence to the MEP < 50% were [basal 25 vs. 12 weeks 16] and participants with adherence to the MEP ≥ 50% were [basal 35 vs. 12 weeks 33]. Although the plots show the information visually, the full numerical data are detailed below. LLS: overall participants [basal 13 (11–14.5) vs. 12 weeks 14 (12–16), *p* = 0.007] participants with adherence <50% [basal 13 (12–14) vs. 12 weeks 13 (10.5–15), *p* = 0.65]; participants with adherence ≥50% [basal 13 (11–16) vs. 12 weeks 15 (13–16), *p* = 0.005]. ULS: overall participants [basal 13 (12–15) vs. 12 weeks 15 (14–18), *p* = 0.001] participants with adherence <50% [basal 13 (12–14) vs. 12 weeks 14 (12–14.5), *p* = 0.002]; participants with adherence ≥50% [basal 13 (13–15) vs. 12 weeks 16 (15–19), *p* = 0.001]. AE: overall participants [basal 59 (50–68.5) vs. 12 weeks 61.5 (56–79), *p* = 0.001] participants with adherence <50% [basal 56 (42–63) vs. 12 weeks 58 (49–61), *p* = 0.09]; participants with adherence ≥50% [basal 62 (55–71) vs. 12 weeks 66 (58–80), *p* = 0.005].

The 12-week MEP also improved the physical performance, interpreted as an increase in median lower (Figure 3A) and upper extremity strength (Figure 3B), and aerobic endurance (Figure 3C). Significant differences were only observed in participants with an adherence ≥50% to the MEP regardless of their HIV status as described in Figure 3. Improvement in agility was found but only in HIV-negative participants [TUG basal 7.31 s (6.25–8.45) vs. TUG 12 weeks 6.23 s (5.63–7.02), *p* = 0.001]. No differences were found for balance, flexibility, or physical function.

**Figure 4:**
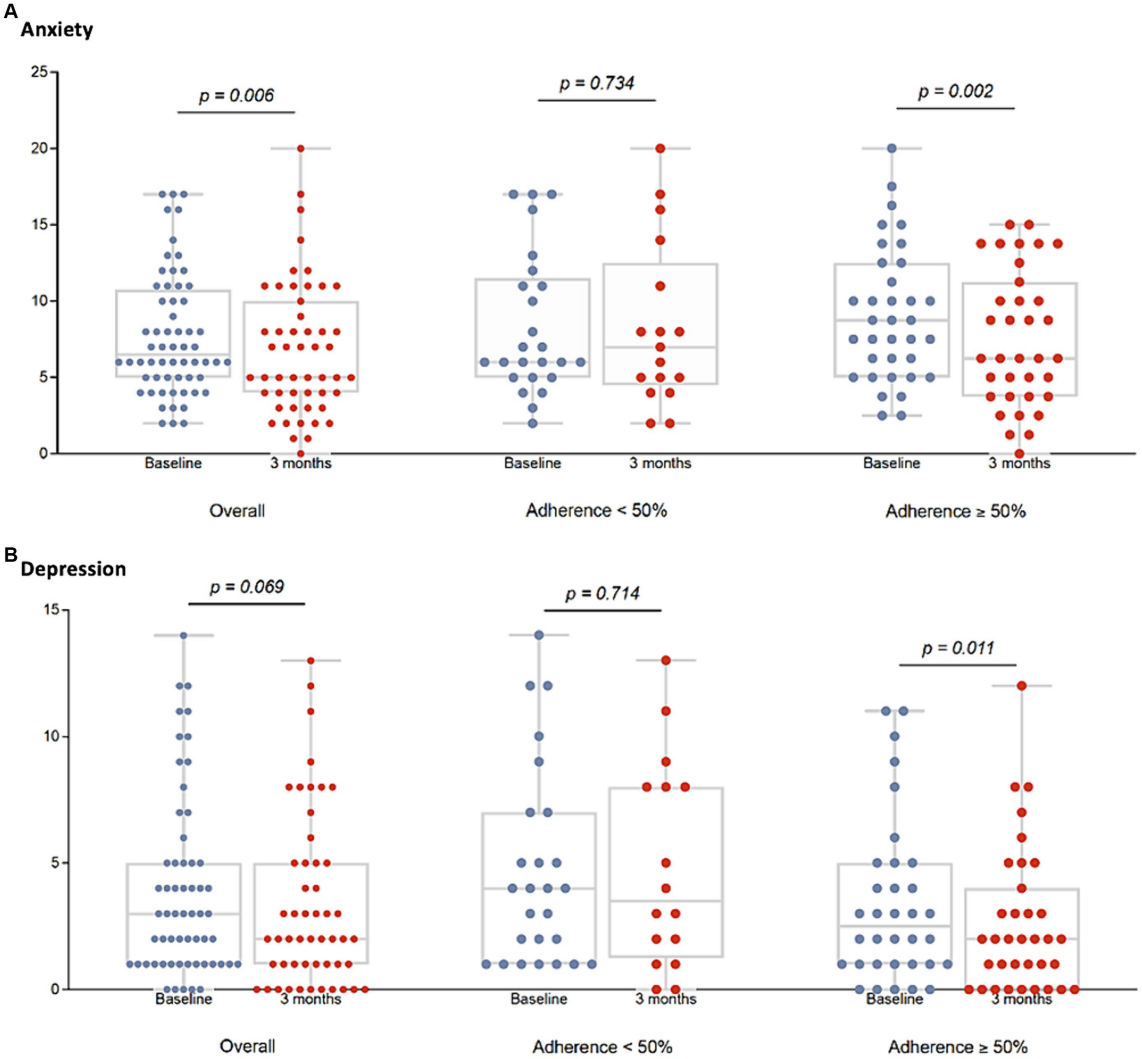
Effect of the 12-week MEP on anxiety and depression by adherence to the MEP on older adults regardless of HIV status. Anxiety was evaluated using the HADS, and depression through the GDS-SF. Anxiety **(A)** and depression **(B)** were represented by dot plots at the baseline visit and after the 12-week MEP, in the overall population, and by adherence to the MEP. Total number of participants were [basal 60 vs. 12 weeks 51]. Participants with adherence to the MEP < 50% were [basal 25 vs. 12 weeks 16] and participants with adherence to the MEP ≥ 50% were [basal 35 vs. 12 weeks 35]. Although the plots show the information visually, the full numerical data are detailed below. Anxiety: overall participants [basal 6.5 (5–10.5) vs. 12 weeks 4 (4–10), *p* = 0.006] participants with adherence <50% [basal 6 (5–11) vs. 12 weeks 7.5 (4.5–12.5), *p* = 0.73]; participants with adherence ≥50% [basal 7 (4–10) vs. 12 weeks 5 (3–9), *p* = 0.002]. Depression: overall participants [basal 3 (1–5) vs. 12 weeks 2 (1–5), *p* = 0.06] participants with adherence <50% [basal 4 (1–7) vs. 12 weeks 3.5 (1.5–8), *p* = 0.71]; participants with adherence ≥50% [basal 2.5 (1–5) vs. 12 weeks 2 (0–4), *p* = 0.01].

**Figure 5:**
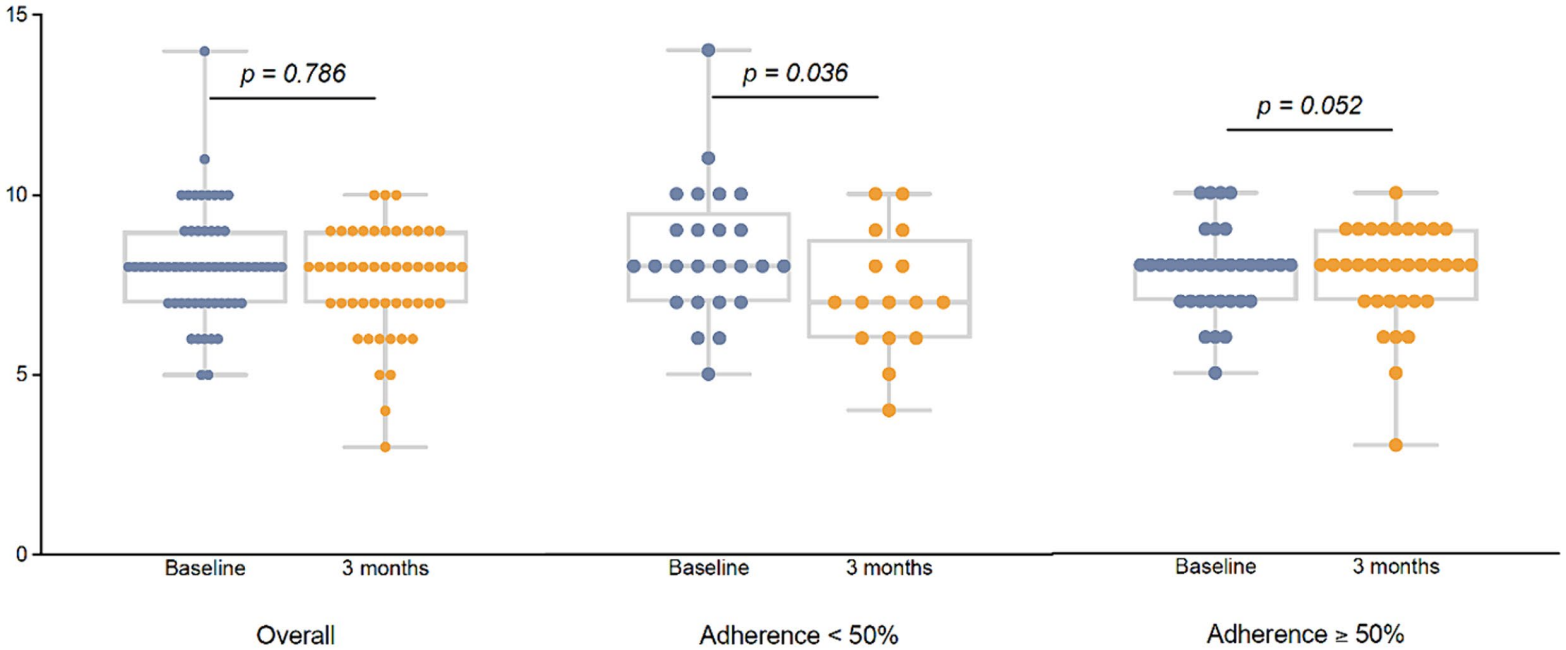
Effect of the 12-week MEP on muscle mass by adherence to the MEP on older adults regardless of HIV status. Muscle mass was measured using the ASMI by electrical bioimpedance (Tanita BC-418^®^), and it was represented by dot plots at the baseline visit and after the 12-week MEP, in the overall population, and by adherence to the MEP. Total number of participants were [basal 60 vs. 12 weeks 51]. Participants with adherence to the MEP < 50% were [basal 25 vs. 12 weeks 16] and participants with adherence to the MEP ≥ 50% were [basal 35 vs. 12 weeks 35]. Although the plots show the information visually, the full numerical data are detailed below. Overall participants [basal 7.92 (7.20–8.81) vs. 12 weeks 7.92 (7.01–8.52), *p* = 0.78] participants with adherence <50% [basal 8.35 (7.44–9.26) vs. 12 weeks 7.09 (6.08–8.62), *p* = 0.03]; participants with adherence ≥50% [basal 7.88 (7.13–8.38) vs. 12 weeks 8.24 (7.09–8.52), *p* = 0.05].

Significant improvement in the HADS anxiety scale (Figure 4A) and GDS-SF (Figure 4B) was found only in those participants with an adherence ≥50% to the MEP regardless of their HIV status as shown in Figure 4. No differences were found regarding the HADS depression scale, falls, or risk of malnutrition. Significant improvement in quality of life was also found, but it was only related to physical health (WHOQOL-HIV-BREF) [basal 10.5 (9–12) vs. 12 weeks 10 (9–12), *p* = 0.001]. PA significantly increased after the 12-week MEP irrespective of HIV status and adherence [basal 396 METS (239.2–990) vs. 12 weeks 1746 METS (1039.5–2,619), *p* = 0.001]. We observed a slight worsening in ASMI only in those participants with an adherence ≤50% to the MEP regardless of their HIV status. As shown in Figure 5, participants with good adherence did not experience an improvement.

## 4 Discussion

Our results demonstrate that a 12-week MEP enhances frailty by increasing robustness in OAWH, and improves physical performance, mood status, and preserves muscle mass in older adults regardless of HIV status. In our study, out of the four frail participants with HIV at baseline, two transitioned from frail to prefrail and one to robust but further studies are needed to assert the ability of an exercise program to reverse frailty in OAWH. On the other hand, more than half of the non-robust participants in our study became robust after the 12-week MEP which makes the results particularly significant. The interest and scientific evidence on frailty and HIV have grown markedly over the past 10 years, but the bulk of the published material has focused on the prevalence of frailty in different countries and settings (20), on its ability to predict adverse health events (21), on its relationship with biomarkers (22, 23), and/or with other health conditions (24), nevertheless, until now, no evidence has been available concerning treatment. Some scientific societies recommend frailty screening in people over 50 years of age with HIV (25), however, health professionals had no evidence-based intervention to offer until now beyond extrapolating on the interventions that have proven effective in the overall population (6). Our study shows the efficacy of a personalized MEP performed in real life conditions that can be easily prescribed to OAWH in daily practice; the complete MOVIhNG MEP is now freely available on the MOVIhNG App, both on Apple and Android. Controversy exists as to whether center- or home-based exercise programs are better. In this regard, it seems crucial to consider that the main objective should be for OAWH to integrate exercise into their daily life as a habit since its beneficial effects persist as long as it is maintained. Randomized controlled trials are highly supervised, and the adherence to center-based exercise programs is consequently high during the studies. However, many times they are quite difficult to maintain in the long-term, especially in older adults, due to some possible barriers such as economic issues, distance from home, and the uncomfortable feeling of being in a group for any reason (26, 27). Only exercise programs that consider the preferences and characteristics of participants can become a habit (28). Deserves to be highlighted that the dropout rate in our study at 12 weeks was 15%. According to the available scientific evidence, this is not a high dropout percentage; in fact, it is below what is described in RCTs – around 20% – both in studies with older adults from the general population (9, 29, 30) and in the few available studies in older adults with HIV which are also RCTs (31, 32). Considering that we are comparing the losses from a home-based program with controlled clinical trials, we can affirm that the expected loss percentage would be higher, and therefore, it would be a strength of our program.

In our study, participants adhering to the program achieved a very high percentage of robustness, but, interestingly, among those with poor adherence to the MEP, the percentage of robustness increased as well. This highlights the fact that some exercise is better than none but that there must be a concrete exercise pattern with prescribed dose, type, frequency, and intensity for that to be possible (33). Only one-third of adults in the overall population meet international guidelines for combined aerobic and resistance training and older age is associated with less compliance with the guidelines (4). The lack of information, accessible tools, and specific objectives adapted to the needs of each individual may be the reason for the poor adherence to the recommendations. Our results have demonstrated that the 12-week MEP has succeeded in changing the routine, significantly increasing PA, multiplying the PA performed by the participants in METS by four after completing the program, irrespective of HIV status and adherence. The latest WHO guidelines state that “all activities count,” underlying the health value of physical activity sessions lasting <10 min (11), and researches have recently published the health-related potential of promoting 2–3 short bouts of higher intensity PA when integrated into the daily living activity even if it is not a structured exercise program that emphasizes the importance and effectiveness of integrating exercise into a daily routine (34).

A scoping review focused on PA and exercise for OAWH shows that exercise enhances physical, mental, and functional status among OAWH with no adverse effects despite the shortage of solid evidence (35). Recently, a meta-analysis including seven randomized controlled trials showed that PA interventions for OAWH are effective for the improvement of walking capacity but not for cardiorespiratory fitness, body composition and weight, or psychological profiles, probably due to the variability of the interventions (36).

Our 12-week MEP improved physical performance, upper and lower extremity strength, and aerobic endurance, in participants with an adherence ≥50% to the MEP. Other exercise programs have previously found the benefits of exercise in OAWH in different measures. Thus, a 1 year supervised resistance exercise program focused on participants 60 years or over who demonstrated improvements in both muscular strength and physical fitness (37); a 24-week supervised combined cardiovascular and resistance exercise intervention in adults over 50 years significantly improved strength and endurance (31), and a 24-week supervised community-based exercise program including people 18 years or over with HIV (50% were older than 50 years) showed potential improvements on strength, flexibility, and self-reported physical activity (38). All three were center-based studies and none recruited frail participants.

In addition, we found significant improvements in anxiety and depression symptoms after the 12-week MEP but only in those participants with an adherence ≥50% to the MEP. Various PA interventions have demonstrated their effectiveness in improving symptoms of depression and anxiety, and adults with HIV are among those in which the benefits are greater from a wide range of diseases and clinical conditions (39). We observed a slight worsening in the ASMI, but only in those participants with an adherence ≤50% to the MEP independently of HIV status, whereas participants with good adherence did not experience an improvement. A meta-analysis of 13 randomized controlled trials designed to determine the effects of resistance training upon muscle strength and muscle mass in people with HIV, without focusing on OAWH, concluded that resistance training appears to be effective in improving muscular strength but not muscle mass (40), and a subsequent work showed that resistance training preserves muscle mass in OAWH but does not improve it (41). These findings are congruent with those reported within the overall older population in various meta-analysis and systematic reviews (7, 42). Therefore, the relevant contribution of our study regarding muscle mass is to show the loss of muscle mass in those who do not comply with the exercise program, which refers to the risk of becoming sarcopenic or frail OAWH due to not exercising with the passage of time.

Some limitations of our study should be considered. The first one is that this is not a randomized controlled trial, which is considered the gold standard for evaluating the efficacy of an intervention. In our opinion, this limitation has become a strength because our goal was to conduct a study in which the exercise intervention under real-life conditions could demonstrate benefits and facilitate participants’ adherence to a simple exercise program as controlled interventions in the clinical trial setting are not always easily replicable. The second limitation is the method we used to assess adherence to the MEP. Due to the very nature of the study design, adherence was assessed using the self-reported method. We implemented various tools (e.g., a weekly activity diary on the website, steps taken through the bracelet) to overcome this possible limitation, and the different results obtained after the 12-week MEP according to adherence support the method.

In conclusion, a 12-week MEP performed in real life conditions is capable of enhancing frailty by increasing robustness in OAWH. This program has also been demonstrated to improve physical performance, mood status, and quality of life and to preserve muscle mass in older adults independently of HIV status. Given the scientific evidence, certain authors have raised ethical concerns about the decision not to prescribe physical exercise when frailty is identified in older adults (43). Our results provide scientific evidence in OAWH, and offer a MEP easily prescribed in daily clinical practice; thus, there is no reason not to prescribe a MEP in OAWH, especially in frail OAWH.

## Data availability statement

Data will be available by the corresponding author to any interested researcher who submits a proposal. Request for access to MOVIhNG data should be sent to fbranas@gmail.com or msconde@gmail.com. The study protocol and informed consent will be provided if required.

## Ethics statement

The study was approved by the Medical Research Ethics Committee Hospital Universitario Ramón y Cajal and Medical Research Ethics Committee Hospital Universitario Infanta Leonor in Madrid (Spain) ceic.hrc@salud.madrid.org. The study was conducted in accordance with the local legislation and institutional requirements. The participants provided their written informed consent to participate in this study.

## Author contributions

FB: Conceptualization, Data curation, Formal analysis, Funding acquisition, Investigation, Methodology, Project administration, Resources, Supervision, Validation, Visualization, Writing – original draft, Writing – review & editing. JD-Á: Data curation, Formal analysis, Writing – original draft, Writing – review & editing. JF-L: Conceptualization, Data curation, Writing – original draft, Writing – review & editing. BV-B: Data curation, Writing – original draft, Writing – review & editing. RG-M: Conceptualization, Writing – original draft, Writing – review & editing. EM: Data curation, Writing – original draft, Writing – review & editing. PR: Data curation, Writing – original draft, Writing – review & editing. JM-S: Formal analysis, Writing – original draft, Writing – review & editing. LL: Data curation, Writing – original draft, Writing – review & editing. MM: Data curation, Writing – original draft, Writing – review & editing. FD: Data curation, Writing – original draft, Writing – review & editing. MS-C: Conceptualization, Data curation, Formal analysis, Funding acquisition, Investigation, Methodology, Project administration, Resources, Supervision, Validation, Visualization, Writing – original draft, Writing – review & editing.
